# Research for food and health in Europe: themes, needs and proposals

**DOI:** 10.1186/1478-4505-9-37

**Published:** 2011-09-29

**Authors:** Mark McCarthy, Amina Aitsi-Selmi, Diána Bánáti, Lynn Frewer, Vasant Hirani, Tim Lobstein, Brian McKenna, Zenab Mulla, Giulia Rabozzi, Raluca Sfetcu, Rachel Newton

**Affiliations:** 1 Department of Epidemiology and Public Health, University College London, London, UK; 2 Central Food Research Institute, Budapest, Hungary; 3 Centre for Rural Economy, Newcastle University, Newcastle, UK; 4 International Association for the Study of Obesity, London, UK; 5 Emeritus Professor, University College Dublin, Dublin, Ireland; 6 University of Study, Milan, Italy; 7 Medical Research Council Unit of Lifelong Health and Ageing, London, UK; 8 Independent consultant, Bucharest, Romania; 9 Sociedade Portuguesa de Inovação, Porto, Portugal

## Abstract

**Background:**

Diet, in addition to tobacco, alcohol and physical exercise, is a major factor contributing to chronic diseases in Europe. There is a pressing need for multidisciplinary research to promote healthier food choices and better diets. Food and Health Research in Europe (FAHRE) is a collaborative project commissioned by the European Union. Among its tasks is the description of national research systems for food and health and, in work reported here, the identification of strengths and gaps in the European research base.

**Methods:**

A typology of nine research themes was developed, spanning food, society, health and research structures. Experts were selected through the FAHRE partners, with balance for individual characteristics, and reported using a standardised template.

**Results:**

Countries usually commission research on food, and on health, separately: few countries have combined research strategies or programmes. Food and health are also strongly independent fields within the European Commission's research programmes. Research programmes have supported food and bio-technology, food safety, epidemiological research, and nutritional surveillance; but there has been less research into personal behaviour and very little on environmental influences on food choices - in the retail and marketing industries, policy, and regulation. The research is mainly sited within universities and research institutes: there is relatively little published research contribution from industry.

**Discussion:**

National food policies, based on epidemiological evidence and endorsed by the World Health Organisation, recommend major changes in food intake to meet the challenge of chronic diseases. Biomedical and biotechnology research, in areas such as 'nutrio-genomics', 'individualised' diets, 'functional' foods and 'nutri-pharmaceuticals' appear likely to yield less health benefit, and less return on public investment, than research on population-level interventions to influence dietary patterns: for example policies to reduce population consumption of trans fats, saturated fats, salt and energy density. Research should now address how macro-diets, rather than micro-nutritional content, can be improved for beneficial impacts on health, and should evaluate the impact of market changes and policy interventions, including regulation, to improve public health.

**Conclusions:**

European and national research on food and health should have social as well as commercial benefits. Strategies and policies should be developed between ministries of health and national research funding agencies. Collaboration between member states in the European Union can yield better innovation and greater competitive advantage.

## Background

'A major issue for public research is targeting prevention of diet-related diseases. The major chronic diseases are caused substantially through poor diets. Moreover, the dramatic rise in overweight and obesity is also predominantly due to worsening diet across the EU. These trends will have a negative impact on life expectancy in the EU and reduce quality of life.' [1]

In Europe, four proximal behavioural factors - diet, tobacco, lack of physical exercise and alcohol - are major contributary causes of chronic diseases [2]. The World Health Organisation has sought to raise awareness and develop policy on food-related health challenges in European Member States through capacity building, monitoring trends, and reporting on services and strategies. WHO proposes that: "Research should support a better understanding of the role of nutrition, food safety and lifestyle factors in disease development, provide information on risk factors and determinants throughout the whole food supply chain, and strengthen the evidence base for, and the health impact of, interventions and policies." [3]

Support for agriculture has been a cornerstone of policy for European integration since the Treaty of Rome in 1957. The food sector is the second largest manufacturing sector in Europe, currently with a turnover of over €800 bn and 4 million employees [1], and the food retail and catering sector together is as large. 'Health', in contrast, is a relative newcomer for European policy. Public health (disease prevention and health promotion) was recognised within the EU Treaty in 1992, but health services are the primary responsibility of member states. Agriculture, food and health are significant themes in the European Union's current Seventh Framework Research Programme (FP7). Food and health have been considered together within the Food theme of FP7, but the research supported has been mainly for production of food, including biotechnology, and its microbiological and chemical safety [4]. Health itself is a major theme in FP7, but almost all the funding has been directed to biotechnology and biomedicine, and very little to broader social research for health [5].

In the year 2000, the EU's Lisbon Strategy proposed coordination and increased competition within the European Research Area to promote economic and social improvement. In the EU's Sixth Framework Research Programme (2002-2006) a European Technology Platform 'Food for Life' was created for food industry research, and the Platform's report [6] contributed to the inclusion of the sub-theme 'Food and Health' within the Knowledge-Based Bio-Economy theme of the Seventh Framework Research Programme (2007-2013) The European Commission has also developed Joint Programming Initiatives for coordination of research programmes between member states, and in 2009 the topic 'Healthy Food for a Healthy Life' was chosen as one of the first of these new instruments [7]. The European Union now has a new overarching strategy for the period 2014-2020, with seven Flagship themes, and research is incorporated within the theme of Innovation for economic and social improvement [8].

FAHRE (Food and Health Research in Europe) [9] is a collaborative project created in response to a call from the European Union's food and health research programme for 2009. The call sought to deepen knowledge of national research on food and health and contribute to planning and coordination of future research, FAHRE has four activities: to map national food and health research and research structures; to determine strengths and gaps at European level; to develop proposals for improved European performance in food and health research; and to disseminate the findings in support of the European Research Area. These were phased sequentially, so that first there was mapping in member states, next there was analysis and synthesis to European level, then development of proposals and finally dissemination to targeted and more general audiences. FAHRE has seven partners contributing to these activities, with the first two activities also supported by information from specialist experts. The present review combines the expert analyses undertaken for FAHRE's second activity.

## Methods

**Figure 1:**
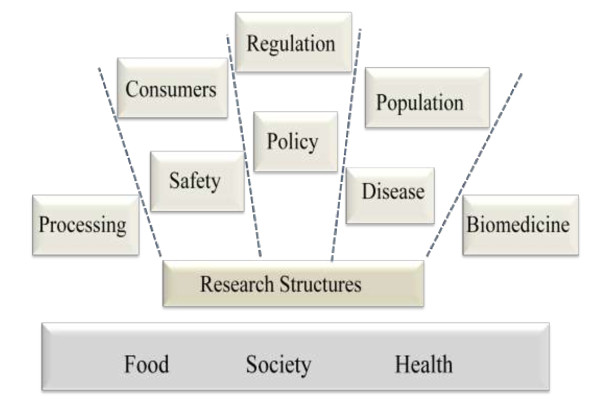
**FAHRE food and health research typology**.

The initial task was to determine the themes covered by 'food and health': the range is indicated in Figure 1. Agricultural research has a long tradition - for example, as well as genetic husbandry, it was important for environmental experiments, and in developing statistical analysis - but agriculture's direct effects on dietary health are not well understood. Converting food into food products (processing) creates issues of content, taste and safety, while the retail and catering industries are concerned with supply and choice. In different ways, primary production, processing and retail industries affect consumer choice and are subject to forms of policy and regulation. Food impacts on the population and its constituent groups, and understanding of its effects on health and disease have come both through population (epidemiology) as well as laboratory studies. Clinical research (patients with diseases) addresses some conditions where there are evident precipitating causes, such as allergies, while laboratory research is investigating the mechanisms at tissue, cell and molecular level. Added to this, studies seek to connect genetic characteristics with food exposures and intervening factors.

Another objective was to span research employing the natural sciences, including biology and chemistry, the clinical sciences based on diseases, and the social sciences placing the human in social structures and environments. These were identified for the definition for food and health research adopted by the project, but it was understood that multiple disciplines are increasingly used across the thematic sectors.

'Food and health research' for this report refers to research about the production, marketing, choice, regulation and policy for food as it affects health, and the mechanisms and control of diet-related diseases, nutrition and obesity. It covers positive and negative impacts of food on health as well as issues related to under and over consumption of food (undernutrition and obesity). It draws on a wide range of disciplines including psychological, social, management and political sciences, laboratory sciences, clinical medicine, environment and epidemiology, and is undertaken by public, independent and industry organisations.

FAHRE commissioned experts to report on the nine themes forming a spectrum across food, society and health, joining both core disciplines and multidisciplinary approaches. An earlier typology had been developed by the Joint Technology Platform, Food for Life [7]. In addition, for the first activity of FAHRE, a typology of four categories had been developed to classify research programmes. These two further typologies are given in Table 1 to show their overlap and similarities - an estimate of validity for the categories chosen.

**Table 1:** Comparison of fields identified by FAHRE thematic areas experts, by FAHRE country reports and by Joint Programming Initiative (JPI) interim report.

FAHRE Research thematic areas	FAHRE Country Report Programme research fields	JPI interim report
Food production and products Food safety	Production field: design of food (components...), its preparation (processes...), its manufacturing and also home cooking matters linked to health and disease, interface between industry and the scientific sector.	Diet and food production: Establish reliable data on consumer food preferences and acceptance in order to develop new food products and to redesign how foods are produced. Enable redesign and optimisation of food processing and packaging. Foods must always be safe and should be produced in a sustainable way.

Regulation, claims, and food policy for health Consumer behaviour and what influences it	Policy field: regulation (labeling, salt, sugar and fat contents, claims...) and consumers, which will have an impact on diet and therefore on health. It could also focus on programmes more economically oriented, linked to marketing, participation, public expression and access.	Lifestyle: Consumer behaviour and food choice. Understanding of consumer behaviour with regard to food and also to raise consumer understanding of healthy foods and food consumption patterns. Measuring food purchase and consumption behaviour taking into account cultural differences, and subsequently, developing reliable models of consumer choice processes. Effective communication strategies with consumers need to be developed to induce behavioural change directed to improving consumer health and social responsibility.

Population surveys causes and control Health policy for food, nutrition, diet and obesity	Population field: at human and population level, epidemiologic approaches (including biological, social and psychological determinants) and observational and interventional research on behaviours that can explain eating disorders leading to chronic diseases.	Horizontal issues: Mapping food cultures in Europe; research infrastructures (databases, biobanks, food tables); methods, including foresight, to evaluate national or regional programmes; principles for cross-border funding of research.

Food causing disease - excess, imbalance, sensitivity Nutrition micro-elements, malnutrition, gene interactions,	Biomedical field: nutrition and dietary research relating to molecular and clinical aspects, in the pathways and causes of disease, and the mechanisms at different periods of the life course. It could also include food safety, both toxicology and biological.	Chronic diseases: preventing food-related, chronic diseases and increasing the quality of life: 1) understanding of brain function in relation to diet; 2) the effects of diet-gut interaction on intestinal and immune functions; 3) the link between diet and metabolic function (obesity and associated metabolic disorders).

### Work of the experts

Since 'food and health' is a complex field, there is no immediate source to identify experts; and a balance was sought, including geography, gender, experience and employer, as well as field and discipline. The partners in FAHRE were contacted for their suggestions, and a search of the internet was made for relevant organisations. From these enquiries, curriculum vitae were received from potential experts, and the final nine were selected from a short-list with FAHRE partners.

An initial meeting between FAHRE partners led to the development of a template to guide the work of each expert. The template fields included research structures and programmes at national and European levels, and the implications of strengths and gaps for future proposals. The experts came together on two occasions, first to assess and improve the template, and on a second occasion to review progress, and assess barriers in finding results. The experts were asked to use only secondary information. This included the information on national structures and programmes described in the FAHRE country reports. Experts were also asked to use their professional knowledge and data available on the internet to characterise European level activities for their theme. The thematic reports were returned to the lead author who made a synthesis which was the basis of the present review. Details, including webpage URLs and references, are given in the primary reports [10-18].

## Results

### Support at national level

Food processing research funding varies greatly from country to country, with higher levels of it in west Europe and less in the east. There is very little evidence of joint specific funding for this topic anywhere in Europe. Food processing research appears reasonably well funded at EU level, although within larger Food and Health projects. There are research facilities and infrastructure in both western and eastern European countries, with significant facilities in both regions, although these are ageing in the eastern countries. In almost all countries, the industry has projects on food technology diagnostic methods, whereas enforcement programmes are planned by public entities, universities and public research institutes and sometime endorsed by private actors.

The country reports indicated little research on food and health regulation. Some countries had no strategies or national policies for food and health, while others with strategies did not build on the results of food and health policy research - for example, national reports on the health status of the population did not indicate an impact on food and health policies. Governmental and public institutions with an important role in food and health policy were mostly not linked to policy research, and there are very few foresight studies and programmes, which would serve as a good basis for policy planning. In the area of consumers, existing food and health research is mainly in the older EU member states: capacity and capability building is particularly required in Eastern Europe. Research into consumer behaviour and health insufficiently addresses other potential determinants of consumer decision-making (including economic factors, sustainable production, environmental factors and ethical concerns). There is a substantial volume of research relevant to obesity currently being conducted in most countries, and some research on obesity control was related to policy. Six (Denmark, Italy, Latvia, Netherlands, Portugal and Switzerland) of nineteen country reports indicated European collaborative work: five countries are collaborating on an international scale, and four acknowledged plans to do so, or the importance of this type of collaboration.

The majority of countries are undertaking lifestyle and prevention research, or community and population studies and programmes. Finland, Romania, Switzerland and the UK appear to be leaders in the former category, with Lithuania at the forefront in the latter, and the Netherlands and Italy as big players in both. Prospective epidemiological studies and projects are being carried out in most European countries, particularly UK, Germany, Italy and France, but not in Turkey or Romania. Countries with a high level of molecular research such as nutrigenomics included Switzerland, Netherlands, Lithuania, Ireland, France, Finland, Denmark, UK and Czech Republic. Eight of these are involved in the Joint Programming Initiative and together have many research programmes.

### European collaboration

Collaborative research in food and health has been supported by the European Commission within various programmes since the Fourth Research Framework Programme (1994-1998). On the food side, the 2011 programme includes calls for research in fields including satiety control, processed foods with lower salt, fat and sugar content, and food labelling. There has also been much research on food safety, both microbiological and chemical. A large Network of Excellence MoniQA is aiming at validation of methods and standards to analyse foods and food products for safety and quality, while FRISBEE is concerned with food refrigeration innovations for safety (FP7 €8 million). Consumer research is also included in FP7 calls, in projects funded by the Commission's Health Programme, and in foresight work by the European Commission's Joint Research Centre.

In general, research on industry regulation and policy is lacking. However, studies of health promotion are supported by DG Health (e.g. PRO GREENS - Promotion of fruit and vegetable consumption among schoolchildren in Europe), while the International Association for the Study of Obesity (IASO) leads a study jointly with the USA on standards for marketing foods and beverages to children funded through the European External Action Service's Pilot Projects programme.

Europe has led important prospective epidemiological studies linking nutrition, lifestyle and environment, in children, adolescents and older people. Obesity in adults is addressed by EURO-PREVOB and HOPE projects while interventions in children include ENERGY, TOYBOX and EPODE projects. Population-level monitoring research has included European food consumption validation, and the European Nutrition and Health Report providing comparisons of nutritional indicators and health determinants. Child obesity monitoring is being undertaken by a network of countries coordinated by the regional office of the World Health Organization and co-funded by the European Commission. Clinical research on food-related diseases has included intervention studies of single nutrients for diseases including coeliac disease, diabetes and allergies; and using a 'whole-food' approach to address specific disease groups - coeliac disease again, Alzheimers' disease and mental health. Collaborative biomedical research is investigating the foetal origin of degenerative diseases, biological effects of the 'Mediterranean diet', and links between nutrition and genomics.

### Major researcher groups and networks

Food processing technology research is normally concentrated in one or two food research institutes and universities in each country, whereas there are many university departments addressing food safety, (food microbiology and chemical contaminants). An unusual network is PAN Europe, bringing together NGO campaigning organisations working on hazardous chemicals and including consumer, public health, and environmental organisations, trade unions, women's groups and farmer associations, and the SAFE consortium coordinates institutes for science-based food safety policy-setting and regulation. Consumer research appears strongest in the Scandinavian countries, UK, Belgium, the Netherlands and Switzerland, and is represented at European level through BEUC (European Consumers Organisation).

Regulation has been a major concern of the European Food Standards Authority, and equivalent national bodies, it is not a primary research interest. DG Health is concerned with foodstuffs regulation, but mainly animal and plant health, rather than human health. While the European Commission's Directorate for Health and Consumers coordinates a High Level Group on Nutrition and Physical Activity, with representatives from member state governments, and the European Platform on Diet, Physical Activity and Health, bringing together industry and policy stakeholders, there is no committee or network focusing on European food and health policy research.

At population level, EuroFIR (European Food Information Resource) Network is a European Network of Excellence on food composition databanks involving universities, research institutes and SMEs. Significant concern for dietary policies is made by European health groups including the European Heart Network (EHN), and the European CanCer Organisation (ECCO). There is growing engagement of molecular scientists, geneticists and chemists into biomedical fields, including metabolomics and nutrigenetics/nutrigenomics. They seek to increase understanding through research and education, of professionals and the general public, of the role of genetic variation and dietary response, and the role of nutrients in gene expression. Other groupings include the Federation of European Nutrition Societies (FENS), and the industry-funded ILSI (Europe, International Life Sciences Institute) which coordinates the EURRECA Network of Excellence on micronutrient requirements in Europe.

In the private sector, three global companies have strong European research presence: Danone, with 500 staff at Wageningen, The Netherlands and Palaiseau, France; Unilever, with 2500 staff at Port Sunlight, UK, Colworth, UK and Vlaardingen, the Netherlands; and Nestlé at Lausanne, Switzerland. The European Food Information Council (EUFIC), representing both processing and retail industries provides information on food safety and health for the public and professionals with an interest in food.

### Research systems at national levels

ERAWATCH country research profiles describe national research systems, policies, policy support measures, research organisations and key research indicators. European research is predominantly planned and financed by Ministries of Science and/or Education, or equivalent central body. There is often a policy-making council, sometimes in coordination with sectoral ministries, and ministries may also hold research funds directly. Third tier organisations often administer and implement. The research performers are the universities and public research institutes, with the private sector providing a relatively small proportion. Many member states are restructuring the research sector, with smaller Member States expanding the numbers of new universities, and larger states regrouping and consolidating the sector.

Modernisation of the research system in several eastern member states, particularly the larger ones, remains a challenge. However, the EU Structural Funds include substantial funding for research (and innovation), with an emphasis on supporting industry research as well as the public sector. Broadly, the European Regional Development Fund can support capital investments (science parks, laboratories), and the European Social Fund supports people (higher training programmes, exchange).

National agricultural research has been mapped by the Standing Committee for Agricultural Research [19]. FAHRE has reported structures for food and health research in Europe [9]. Structures for funding health research are presented on the web-page of STEPS (Strengthening Engagement in Public Health Research) [20].

### Knowledge needs

Research and innovation in food processing should be both fundamental and consumer-oriented and address the need for food products that promote healthy eating, including the growing older population, and migrants. The food industry is devoting many millions of Euros to laboratory searches for new 'functional' ingredients in foods, often present at very low concentrations. RTD issues for food processing include effective extraction systems, how to place the 'functional' ingredients in food, and methods for keeping them intact through the processing and distribution process. However, there is no lack of nutrients across the population in general, and the value of these products is not supported by evidence. More research is needed to understand what population groups are consuming such products, how overall dietary intakes are affected, and therefore what public health benefits these products are achieving.

Food safety research addresses food pathogens, control of virulence and chemical contaminants, and seeks to develop new systems and methods for control. Current themes include new technical methods of heating and non-thermal microbial inactivation methods (e.g. ohmic heating, pulse electric field, high pressure): non-thermal mechanism better retain nutrients (e.g. vitamins in fruit juices). Much needed safety research is biological, or technical (new food processing and preservation technologies) rather than health research. There should also be more research on human perception of risks and risk communication.

There has been a concerted research effort by the health sector, and the commercial sector with health care interests (including medical equipment, pharmaceuticals and treatment solutions), to create products effective for managing obesity, but with limited success. This has led researchers to turn their attention towards non medical approaches, including food-related health behaviour and the environment (physical, financial, social, educational, regulatory etc). However, while there are several examples of national policy interventions on food marketing and food composition to improve public health in European countries - including the Danish increase in tax on sugared beverages, the French ban on vending machines in schools, Norwegian subsidies given for the distribution of fruit and vegetables, the Finnish Allergy programme with children on specific diets, the UK ban on TV advertising of unhealthy foods to children and the Swiss legal limit on trans-fatty acids in vegetable oils - there has been little evaluative research relating regulation to effects, for example through population surveillance and health impact assessments.

There is increasing research on food-related behaviour, including experimental interventions (for example, information, or community based programmes targeting improved dietary choice): the interaction between biological, psychological and socio-cultural factors is important for dietary choice. Current consumer research tends to relate to a single outcome, such as health, sustainable consumption, or production acceptance: simultaneous comparison of several domains, for example including the risks and benefits to health, the environment, socio-economic factors, and ethical concerns, would be a relevant future research field.

Targets are needed for national food-based dietary guidelines, with interventions at public and industry levels to promote healthy eating. Population studies are of crucial value. There should be evaluation of biomarkers in relation to dietary data, along with studies in nutrigenomics. Better measures are also needed in, for example, quality of life measures which incorporate peoples' experience of life as well as clinical assessments, or the impact of a specific food-related disease on the economic functioning of the individual, the household, and health care services.

Collection of data needs to be maintained over time to monitor changes both in foods and eating habits. Research can investigate food-based interventions for diseases: (a) to promote good function e.g. gut absorption, brain function (b) prevention of disease, e.g. Alzheimer's disease, some cancers, and metabolic syndrome, and (c) improving prognosis during disease, e.g. cardiovascular disease. Bringing about behavioural change is challenging. Health care professionals have used 'motivational interviewing', financial incentives in dieting plans, and cardiovascular genetic risk profiles. However, communicating the results of genetic tests does not appear to result in behaviour change, and weight loss is difficult to maintain.

Biomedical research at molecular-level research has focused on micronutrients, both at individual country level and also cross-European and international levels. But while epidemiological studies always find a link between cardiovascular disease and fruit and vegetable intake, randomised controlled trials with vitamin supplements and with fruit and vegetables have shown no effect on cardiovascular disease. The focus has therefore shifted away from micronutrients towards whole diets/whole foods, for example macronutrients and different dietary patterns such as Mediterranean and Nordic diets.

### Support at European Union level

#### Food research

The European Technology Platform (ETP) "Food for Life" is an industry-led, public/private partnership supported by the European Commission DG Research [6]. Its Strategic Research Agenda in 2007, and Implementation Action Plan, in 2008, proposing proposed "new processes, products and tools that: Improve health, well-being and longevity, build consumer trust in the food chain, and derive from sustainable and ethical production." The ETP is developing a new workprogramme from 2011.

The Seventh Framework Programme Agriculture research theme has included a section "Fork to farm; health and well-being", with topic subjects including

• Consumer, behavioural, societal and cognitive sciences related to food and feed;

• Nutrition and diet-related diseases, including obesity;

• Improved quality and safety, both chemical and microbiological, of food, beverage and feed.

Following a process of consultation, in 2010 the European Commission invited Member States to launch Joint Programming Initiatives in three areas [7]. For 'A Healthy Diet for a Healthy Life', a temporary secretariat has been established in the Netherlands. The permanent governance structure and secretariat is planned from September/October 2011.

#### Health research

The Sixth Framework Research Programme (2002-2006) focused medical research within 'Life Sciences', and gave particularly strong funding to genomics. Broader public health research was put into the 'eighth' Policy strand. The Health call for the Seventh Framework Programme has three pillars, covering basic sciences, applied (clinical) research and public health/health systems. The programmes have covered major disease areas such as cancer, respiratory and neurological diseases as well as cross-specialty themes such as lifecourse, equity and ageing. Food-related diseases are not addressed directly, but food and nutrition are included in research proposals both on the causes and management of diseases, in studies of the determinants of health and in population health interventions.

The European Commission's Directorate for Health and Consumers' EU Platform for Action on Diet, Physical Activity and Health seeks 'to pool Europe's knowledge on what works - and what does not - and to disseminate best practice across the European Union' [21]. The composition of the Platform has included officials, industry and civil society organisations. Five fields for action identified by the Platform members are: Consumer information, including labelling; Education; Physical activity promotion; Marketing and advertising; Composition of foods, availability of healthy food options and portion sizes.

#### Funding

The European Commission calculates that financial support for research on diet, dietary habits and genetic factors totalled about €101 million for five years (1998-2002) in FP5, approximately €178 million for five years (2002-2006) in FP6 and approximately €81 million in the first four years (2007-2010) of FP7 [1]. In FP6 and FP7 approximately €36 million were allocated to projects on allergies, €81 million to prevention of obesity, of which about half was for prevention of obesity in children, and approximately €66 million to identification of bioactive compounds in food and of the mechanisms governing the way they act and to related improved processing. The nutritional needs of an ageing population received €22 million under FP5. €17 million were invested in research projects on consumer needs and behaviour, in FP6 €21 million and in FP7 so far €11 million. The 2010 workplan is addressing production of food for the low-income population.

Other relevant arrangements for European research include:

• The EUREKA programme provides co-funding for research by large companies, and (in the EUREKA Eurostars Programme) by SMEs. EUREKA Umbrellas support research and development in thematic networks, including Euro-Agri in fields of biotechnology, genomics, proteomics and food technology, as well as quality, safety and traceability.

• COST (European Cooperation in Science and Technology) enables coordination of nationally-funded research on a European level. It supports national facilities, institutes, universities and private industry to work jointly on Research and Development in the nine domains, including (but separately) Biomedicine and Molecular Biosciences; Food and Agriculture; and Individuals, Societies, Cultures and Health.

• Research Infrastructures are facilities, resources or services, including, equipment, computing, software and communications, which support the research community in conducting research. An advisory group, the European Strategic Forum for Research Infrastructures (ESFRI) advises on priorities, and there RIs in the health field include networks of clinical research centres and high security laboratories.

• The European Science Foundation (ESF) provides cooperation and collaboration in European research and development, European science policy and science strategy. Its Biological and Medical Sciences Roadmap Working Group has proposed research infrastructures for food and health research.

• SCAR, the Standing Committee on Agriculture and Research, established by the European Commission Directorate, reported on 'health' as one of eight 'driving forces' for agriculture in Europe, focused on food security [22].

## Discussion

### Thematic research areas

#### Processing

The European Union strategy for research and innovation across all sectors seeks to raise investment to the levels of USA and Japan through increasing commercial investment. In the European food industry does not have high levels of investment in research, and the larger companies keep their research private for commercial reasons, so that investment can only be measured as inputs rather than outputs. Several countries have established public research organisations to support SMEs, working principally in new product development, safety and presentation. The marketing and retail industries have separate approaches. There has been collaboration with the pharmaceutical industry on supplements in food, but this will align more with the non-patent industry rather than the more-regulated sectors.

The larger health agenda requires different initiatives with industry. In the Directorate for Health and Consumers' European Platform for Nutrition, Physical Activity and Health, industry has described its contributing activities across areas of food presentation and offer (Table 2). Suggestions were also made for creating networks for the exchange of research findings and techniques, funding professional development in relevant research areas, and providing funding for research.

**Table 2:** Areas of industry action in relation to food and health

Industry area	Action
*Presentation*	Modifying food product labels and/or labelling policies

	Altering nutritional composition - usually levels of fat, sugar or salt

	Changing products - reducing less healthy options, introducing healthier options

	Altering the amount of a food product understood to be, or provided as, a "portion"

	Proposing and/or implementing limits or codes of practice for advertising

*Offer*	Education about nutritional values or healthy diets

	Producing and/or distributing information about nutritional values or healthy diets

	Promoting health qualities of own products

	Changing food purchasing patterns through mechanisms implemented at the point of purchase

	Workplace diet and lifestyle (holistic) programmes

#### Food safety research

Much funding has been given to food safety over the past 15 years, both in the food chain and possible human infections such as BSE. More practically, there has been attention to food handling procedures and standards, and improvements in sterilising foods for retail consumption. Yet much food remains unsafe to consumers because of an excess or imbalance of ingredients in the diet. European and national food safety agencies need an 'epidemiological transition' in food safety research, away from contamination and acute disease, and towards 'safety' that encompasses food that reduces chronic diseases.

#### Consumers

Internationalisation of food supplies, more eating out (and less home-cooking), and growing attention to 'convenience' are all influencing eating habits and deserve close attention. Research must address the balance between commercial opportunity and consumer protection. Food labelling and portion sizes are important on the retail side. Consumer science can contribute to social studies of food causing disease, and the required interventions.

#### Policy and regulation research

A recurrent theme of this review has been the lack of research, including policies and regulations, economic and trade issues, behaviours and marketing, to address the major public health issues of food and health. National and local food policies and cultures are not sufficiently assessed and recorded for changes and commercial or policy interventions. There is lack of understanding of drivers for food access or choice, and especially how to change these - with the engagement of the whole food industry - towards healthier diets. Importantly, social and policy research must be linked into government structures, particularly the ministry of health, so as to integrate with policy and achieve benefits for European citizens.

#### Epidemiology

Europe-wide studies provide an important scale to show the risks and benefits associated with certain diets. There now needs to be more efficient use and updating of existing studies, investigation of whether biomarkers are valid measures of disease outcome, better methods of dietary measurement, and linking of longitudinal studies of individuals with changes in policy, marketing, availability and consumption of foods (e.g. using commercial consumer data).

#### Diseases

Heart disease, cancer and diabetes all relate to diet. The 'epidemic' of adult-onset diabetes is largely due to overeating and consequent overweight. The pharmaceutical industry is seeking to develop 'targeted' nutrients, along the lines of drugs, for controlling disease, but more effective means are needed of reducing the incidence of new disease. New approaches for food allergies could assess why the condition appears to be increasing rapidly, and consider clinical trials of withdrawing ingredients to reveal causes.

#### Food supplementation

Biochemical research has identified increasing detail on components of food, and this has led the food industry into substantial investment in supplementation of foods. The EFSA has criticised the health claims of such foods, as there is little satisfactory evidence that the supplements have any health benefit in this form. This is now leading research away from micro-nutrients towards investigating the impacts of macronutrients and different diets.

#### Genomics

There is very considerable science investment into genomics, which has a broad range of potential impacts. The chronic diseases are associated with multiple gene sites, and the prospect of 'individualised' or 'personalised' diets appears distant as yet, although there may be knowledge gained from certain genetic disorders. Links are needed with epidemiological studies, with sufficient description of exposures.

### Research system needs

#### Infrastructures

Research for food product development requires pilot plant facilities housed in a quasi food factory environment, with drying, freezing, sterilization, membrane processing, packaging, heat treatment and other equipment. Such equipment is currently available, but there is debate on the size of pilot plant required to reflect performance when scaled up to full industrial production size. For food safety research, existing laboratories are able to perform validated analytical techniques and sampling plans for priority chemical contaminants, but there is a need for more rapid methods to apply knowledge, in-line methods for continuous safety management in food processing, and precision/reference techniques for research and confirmatory purposes.

Social science consumer methodologies depend on appropriate information and communication technology infrastructures for analysis to be applied. Qualitative approaches (collection of interview data, running focus groups, ethnographic analysis) may require recording equipment or specific environments (for example, rooms with auditory or video recording equipment). Investigating the organoleptic properties of foods and consumer choices requires the use of sensory panels and specific statistical analysis software. A prerequisite for longitudinal population nutrition and health surveys is sufficient funding for all stages - planning, fieldwork, analysis, interpretation and reporting of the results. Databases need linkage of data with disease and mortality registries. The added value of EU level action lies in the harmonisation of monitoring and surveillance systems to enable evaluation and effective health impact assessment. In the clinical field, there are few institutions focused towards food causes of disease, although allergies are a growing area for collaboration. Laboratory studies, biomarkers and nutrigenomics are being supported in new member states through European regional grants.

#### Structures

Few countries in Europe have a strategic approach to food and health research, and member states know little about each other's programmes. There is a need for more and better multidisciplinary and comparative research, particularly in countries with where traditional food and medical research has not sufficiently incorporated social science approaches. Career paths for interdisciplinary scientists should be supported, for example through greater recognition in national or institutional research assessments. There should be more systematic recording of the relevant research areas and actions, and better use of national and European projects to determine clearly the main research issues.

A Europe-wide consultative forum, physical or virtual, is needed. Models include the Standing Committee for Agricultural, the committees of the European Science Foundation, or the collective actions in ERA-nets. There also could be virtual collaborations, in association with a host institution which could be responsible for communication on relevant research outputs, conferencing, links with policy-makers for knowledge translation, and training and capacity building.

European universities and research institutions need to provide world class research to attract students and researchers; and they need to open up to business and international collaboration to access new funds. Technical equipment has been well provided, through European and national grant agencies, and there is now a need for more "soft" approaches, including training, capacity-building, financial incentives, networks. European data bases are also needed - of experts across different disciplinary fields, of available European equipment, and 'warehousing' data sets for secondary analysis. There is a lack of postgraduate training and courses for research scientists across food and health research, requiring investment in social as well as technological sciences, and in university infrastructures as well as research institutes.

#### Programmes

Much food and health research in Europe is conducted in public institutions. The FAHRE country reports show the volume of work being undertaken and the large number of institutions involved. However, there is an imbalance in the research being undertaken. Most food research has addressed food processing technologies and safety, with less on behaviour, regulation and policy. Similarly, on the health side there has been an emphasis on biotechnology and medical aspects, and less on public-health and policy issues. Given national, regional and local differences in diets, family structures, and genetic factors, more attention to collaborative intervention studies between European partners is needed, perhaps jointly funded by national programmes.

Much technical research has addressed introducing micro-nutrient supplements into foods, but the health benefits are often not established, and claims in products have been challenged by regulatory authorities in both Europe and USA. Exploration of genetic profiles for 'individualised' diets has also been unsuccessful. In contrast, there has been less research on the impact of the rapid growth of retail supermarkets, or the greater use of 'convenience' foods and '24 hour shops'.

Research on barriers to adopting healthier diets should address habits, socio-cultural pressures (e.g. peer group pressures in adolescence) and access to healthy food, or information about healthy eating. Insights from risk-benefit perception and food safety may be relevant. National and regional surveillance of food intake and its determinants remains important. Food composition (including formulation and portion sizes), food marketing (including labelling, packaging and advertising), food behaviour (including factors influencing choice) and food accessibility (including pricing, supply and location) are particularly poorly researched. Methodological research should include standardised approaches to assessing the impact (such as quality of life, economic functioning of individuals and households, health service use, and perceived wellbeing), survey measurement of dietary choices, and psychological aspects (nutritional knowledge, attitudes towards healthy eating, risk-benefit perceptions associated with specific food choices, and standardized behavioural indicators). Foresight activities can be encouraged in order to serve policy-building and decision-making activities.

#### Impacts

Policy-related research should take into account the needs and priorities of inter-governmental agencies (WHO, Council of Europe, OECD), civil society organisations and policy-makers. The research should be framed to have maximum impact, presenting clear and relevant policy options in a lively way that strikes a chord with both decision makers and the public while not ignoring the complexities and uncertainties that characterise the real world of research. Research priorities can be assisted through policy analyses and the development of more coherent links between policy-makers and the research community. Public health professional bodies and advocacy organisations monitor policy developments and can provide strategic commentary on these.

A research question of critical importance is what incentives can be created to help companies move their sales from less healthful products, and encourage companies to invest in the supply of healthy products. StanMark has monitored development of standards for marketing of food and drinks to children across Europe [23]. The wider policy picture also needs research support, from public or public-private funding: for example, research on the most effective use of the public sector's food purchasing power, the use of taxation and subsidy, the use of agricultural policies and industrial investment policies, retail planning policies and economic stimulation policies - directed towards public health benefits. Conflicts of interest undermine the advancement of public-private partnerships, and possible solutions may be suggested, such as a blind trust into which industry and foundation support can be placed, or a form of research levy taken from the commercial operators who benefit from the research.

Developing effective translation of research outcomes into policy must to be prioritised, as well as ways to promote stakeholder and public engagement for research governance and policy development. Bridging the gap between research and policy translation - implementation research - is largely absent from national funding agendas. There needs to be a full range of research disciplines and applications in national funding programmes as well as EU funded initiatives. There should be a set of "standard operating procedures" for national data collection relevant to food and health, comparison of achievement across time and space, and measuring health impacts, including socio-economic variations in population health. Methods of evaluating the health impacts of policies before they are introduced also need to be developed and refined. Besides health impact assessment other approaches (e.g. regular inter-sectoral health forums, networks etc.) could be adapted to the specific policy context.

#### Coordination

WHO is linking member states in action plans for different nutrition themes, including salt, obesity and school meals [24]. Nutrition Action Plans have been agreed by every EU Member State. The European Commission's Joint Programming Initiative [7] will address coordination of food and health research between participating European countries. There is a need for tools to track and share data and disseminate the results in order to prevent duplication of efforts and to encourage synergies.

A listing of resources (skills and equipment) should be made to facilitate research pooling across the European research area. There can also be stakeholder involvement in the development of research requirement portfolios, including relevant industries, scientific disciplines, and consumer organisations across EU member states. Country financial information is also limited. Few countries identify specific amounts available under certain calls for research or as budgets awarded for projects, which makes the evaluation of the cost-effectiveness of research very difficult. There is a need to build clear information systems containing financial information, and to relate it effectively to fields and sub-fields of food and health research.

The contribution of public-private partnerships needs more attention. A few global companies have research divisions in European counties, providing an important technical base for product development. For smaller companies, research is often more linked to the solution of an immediate industrial problem rather than the implementation of a good research programme. The value of emphasising intellectual property rights and patents may be questioned in this field. Many companies are developing open innovation approaches to R&D, combining in-house and external resources to maximize economic value from their intellectual property, even when it is not directly linked to their core business. They have begun to recognise public research as a strategic resource. In parallel, research institutions need to play a more active role in their relationship with industry in order to maximize the use of the research results. Joint funding for research and training programmes and industry support of university degree programmes that meet industry needs, can be a way forward.

Health-related civil society organisations involved in food-related issues (heart disease, diabetes, cancers, nutrition etc) can provide valuable contributions on policy needs and the gaps in the evidence base. More effort could be made to involve them in the development of research funding strategies - including fund-raising. Effective policy translation, with stakeholder and public engagement, of research into outcomes needs also to be prioritised.

## Conclusions

This synthesis of FAHRE Thematic Expert's reports provides information on research programmes, national structures and projects at European level, and gaps and overlaps for the research. Food is an important cause of ill-health in Europe, but the two research fields of food and health are not well linked, at either the national or at European levels. There have been major research programmes on food safety, and epidemiological studies and population surveys have contributed to understanding the trends and causes of disease. However, industry research has focused on technology for nutrient enhancement of processed food, while national research has failed to invest in social, behavioural and policy research on food and ill-health. The findings indicate a move from research on 'healthy food', which concentrates on food products, to research for 'healthy eating' which is concerned with appropriate intake and reducing risks to European citizens.

FAHRE has addressed broad needs and gaps in both research and the structures supporting research. The 'Europe 2020' strategy of the European Union [8] seeks to build economic and social improvement on the base of innovation and research. Research on food and health can contribute by identifying solutions and supporting food markets for healthier eating, and thereby extending healthy and productive lives. And there should be benefits from international cooperation. As one expert proposed: "It is not too extreme to suggest that the 27 member states and the EU all have the identical research food agendas and, while the FAHRE country reports have not been detailed at the individual research project level (this would have been impossible), known areas of duplication of effort could be avoided by better integration and organisation."

## Competing interests

Dr Banati declares her interests as:

• Chairpersons of EU Agencies, Chair (2011-)

• European Food Safety Authority (EFSA), Management Board, member (2006-2010, 2010-2014); Vice-Chair (2006-2008); Chair (2008-2010, 2010-)

• European Commission, European Group on Ethics in Science and New Technologies (EGE), member (2005-2010)

• European Academy of Nutritional Sciences, member

• European Association for Food Safety (EAFS), Executive Board, member (2005-), Vice Chair (2006-2009)

• European Programme Planning Committee of the Toxicology Forum, member(2006-2010)

• FAO/WHO Codex Alimentarius Task Force on the Safety of Foods Derived from Modern Biotechnology, expert (2005-2008)

• ILSI Europe (International Life Sciences Institute), Scientific and Advisory Committee (SAC), member (2003-2006, 2006-2009)

• ILSI Europe (International Life Sciences Institute), Board of Directors (2010)

• International Academy of Food Science and Technology (IAFoST), Fellow (2010-)

• OECD Task Force for the Safety of Novel Foods and Feeds, tag, alelnök (2005-2009)

• The University College of Dublin (UCD) Institute of Food and Health, Scientific Advisory Board, member (2009-).

Other authors declare they have no conflicts of interest.

## Authors' contributions

MM initiated the study, oversaw the data collection, and wrote and revised the final paper; AA-S, DB, VF, VH, TL, BM, ZM, GR, RS contributed equally in collecting data and writing thematic papers, and commented on the final paper; RN contributed to the study design, reviewed thematic papers and commented on the final paper. All authors read and approved the final manuscript.
